# A Study on the COVID-19 Preventive Behaviors of Automobile Manufacturing Workers in South Korea

**DOI:** 10.3390/healthcare10101826

**Published:** 2022-09-21

**Authors:** Ji-Hye Kim, Hye-Young Song, Jin-Hee Park, Purum Kang, Hyun-Ju Lee

**Affiliations:** College of Nursing, Woosuk University, 443 Samnye-ro, Samnye-eup, Wanju-gun 55338, Korea

**Keywords:** COVID-19, risk perception, crisis communication, health literacy, infection prevention behaviors

## Abstract

The present study used a cross-sectional, descriptive survey design to investigate the influencing factors of COVID-19-related infection prevention behaviors of workers in the automobile manufacturing sector. An online survey was conducted on 157 workers in the automobile manufacturing sector of a company in Korea. We analyzed the collected data using SPSS to test whether there were significant differences in COVID-19 risk perception, crisis communication, health literacy, and infection prevention behaviors according to the general characteristics of the participants. An independent sample t-test and a one-way analysis of variance (ANOVA) were performed. A Pearson’s correlation analysis was performed to identify the correlations among COVID-19 risk perception, crisis communication, health literacy, and infection prevention behaviors. Multiple regression analysis was performed to identify the influencing factors of COVID-19 infection prevention behaviors. The regression model was found to be significant, and the employment period at current job, COVID-19 prevention education, source of information, COVID-19 risk perception, crisis communication, and health literacy were also found to be significant. Among the demographic variables, employment period at current job of 5–10 years showed a higher level of infection prevention behaviors than that of <5 years. Moreover, the level of infection prevention behaviors was also significantly higher when COVID-19-related information was acquired through the KDCA/health center. Higher COVID-19 risk perception, crisis communication, and health literacy were associated with significantly higher levels of infection prevention behaviors. Therefore, based on the results, health managers need to develop programs and educate and improve information comprehension and crisis communication skills in order to promote workers’ infection prevention behaviors of emerging infectious diseases in an era of global change.

## 1. Introduction

The World Health Organization (WHO) declared COVID-19 as a global pandemic on 11 March 2020 [1]. According to a report by the WHO, as of 24 June 2022, 539,119,771 people around the world and 18,305,783 people in Korea had been infected with COVID-19, while the mortality rate of COVID-19 was reported to be approximately 0.13% [2]. As of 24 June 2022, the number of confirmed COVID-19 cases in Korea has been on a decreasing trend, but the average daily number of confirmed cases remains high at 7062 cases [3]. The symptoms patients experience are generally mild and dissipate on their own. However, the elderly and patients with pre-existing conditions have a high risk of complications associated with COVID-19 [1,2]. Variants of concern with higher infectivity and disease severity than the original SARS-CoV-2 virus have continued to be identified, and thus compliance with personal infection prevention behaviors is an essential element of the response system for mitigating the spread of COVID-19 within the community [4,5,6]. The WHO and health authorities in each country have been emphasizing infection prevention behaviors, such as social distancing, hand washing, and mask-wearing, as key solutions for slowing the spread of COVID-19 [2,3].

Meanwhile, empirical evidence on cognitive, psychological, and social factors related to health behaviors is needed to increase the practice of preventive behaviors by individuals. A representative theory explaining human health behavior is the health belief model (HBM) [7]. According to HBM, health behavior is affected by cognitive factors, such as perceived threats, self-efficacy, and benefits [7]. Modifying factors, such as demographic variables, psychosocial variables, and behavioral cues, also affect health behavior [7]. In previous studies based on recent HBM, health behaviors (infection prevention behavior, smoking cessation, and osteoporosis prevention behavior) were estimated not only by cognitive factors, such as risk perception, but also by modifying factors, such as health literacy and crisis communication [8,9,10,11,12]. Through this, it can be expected that the prevention of COVID-19 will also be affected by health literacy and crisis communication. Therefore, in this study, risk perception was set as a cognitive factor and health literacy and crisis communication as modifying factors to confirm their roles in the prevention of COVID-19 infection. 

Risk perception, which refers to the subjective assessment that people make about the likelihood of a negative event, such as an injury or a disease, can be identified through the seriousness of the infectious disease or the susceptibility to infection in individuals [12,13]. HBM states that risk perception is an important determinant of health behavior and individuals with a higher risk perception of diseases have higher compliance with health behaviors [7]. Many studies have already confirmed that this is a key determinant of various health behaviors (i.e., health check-ups, self-care behaviors, etc.), as well as preventive behaviors against infectious diseases, such as severe acute respiratory syndrome (SARS), avian flu, and H1N1 influenza (i.e., vaccination, mask-wearing, etc.) [12,14,15,16,17,18].

Crisis communication refers to the process of information exchange and communication about health matters that arise in a dangerous situation [12]. Therefore, accurate communication of infectious disease information in a pandemic situation leads to psychological stability and behavioral change in the public [12]. When individuals intentionally seek health-related information, such behaviors may be influenced by the health awareness and current health status of the individual, while influencing decision-making about health behaviors [8,19]. According to previous studies, the acquisition of sufficient information through intentional information seeking is associated with psychological satisfaction and better health behavior practice [8,20]. Therefore, sufficient crisis communication about infectious diseases can be viewed as an important factor for enhancing the practice of COVID-19 preventive behaviors. 

The importance of health literacy is being emphasized since it has been identified as a key factor related to health behavior and health outcomes [11,21]. Health literacy refers to the motivation, knowledge, and competency needed to access, understand, assess, and apply health information for decision-making related to health management, disease prevention, and health promotion [21]. Health literacy is associated with healthy eating or smoking cessation, while low health literacy was found to be associated with low subjective health perception or high mortality rates [21,22,23]. Recently, the importance of health literacy has also been confirmed in the field of infectious disease control, where low health literacy was associated with low vaccination rates and low literacy about antibiotics [9]. Despite health literacy being identified as a key determinant of health behaviors in various fields, there have been very few studies on health literacy and infectious diseases with high social impact (e.g., COVID-19, tuberculosis, AIDS, etc.), indicating the need for studies on the relationship between COVID-19 and health literacy [9,24].

With community transmission of COVID-19, flexible work arrangements, including working from home and telecommuting, have been actively adopted as a measure to reduce the risk of workplace mass infection in Korea. Unlike office workers, however, workers in the manufacturing sector have jobs that require them to use specific equipment on the production line. In other words, there are limitations to implementing flexible work arrangements, and significant time and effort are needed to improve them. Moreover, individual infections can not only spread in the area where the workplace is located but also to where the workers live and cause community transmission. Although it is very important for such workers to practice infection prevention behaviors in such a public health emergency situation, such as a global pandemic, most studies on COVID-19 that have been reported to date have focused on epidemiology [25], clinical features of infected patients [26], and mental health [12]. Accordingly, the present study focuses on infection prevention activities of manufacturing workers and intends to expand their understanding of them by identifying related factors using the health belief model integrated with health literacy and crisis communication. 

The present study aimed to identify the effects of COVID-19 risk perception, crisis communication, and health literacy on infection prevention behaviors of workers in the manufacturing sector, who are required to work in a face-to-face setting, and to establish basic data for the development of infection control education programs suitable for such workers.

## 2. Materials and Methods

### 2.1. Study Design

The present study used a cross-sectional, descriptive survey design to investigate the influencing factors of COVID-19-related infection prevention behaviors of workers in the automobile manufacturing sector.

### 2.2. Participants

Workers in the automobile manufacturing sector were conveniently sampled for the study in Korea. The sample size was calculated using G*Power 3.1.9.7. Based on regression analysis with moderate effect size of 0.15, two-sided test with significance level of 0.05, and statistical power of 0.80 [27], the minimal sample size needed for 15 predictive variables was calculated to be 139. A total of 160 sets of questionnaires were initially collected, but 157 sets were used in the final analysis after excluding three sets with insufficient responses.

### 2.3. Instruments

#### 2.3.1. General Characteristics

The general characteristics of the participants consisted of sex, age, marital status, education level, employment type, length of employment at current job, subjective economic status, COVID-19 prevention education, source of COVID-19 information, subjective health status, and cohabiting family member with a chronic disease.

#### 2.3.2. COVID-19 Risk Perception 

COVID-19 risk perception was measured using the COVID-19 risk perception tool developed by You [12]. Content validity was verified by 12 infection-medical experts. As a result of the verification, the content validity index (CVI) was 0.82. This tool consisted of two questions: (1) “How likely do you think you are to be infected with COVID-19?” and (2) “How serious do you think the health consequences will be if you become infected with COVID-19?” Each question was rated on a 5-point Likert scale, with higher total scores indicating higher COVID-19 risk perception. The reliability of the tool showed a Cronbach’s α value of 0.720 in the present study.

#### 2.3.3. COVID-19 Crisis Communication 

COVID-19 crisis communication was measured using the tool developed by You [12]. For content validity, it was verified by 12 infection-medical experts, and the CVI was 0.89. This tool consisted of two questions: (1) “Have you obtain enough information about COVID-19?” and (2) “Did you have any difficulty in obtaining information about COVID-19?” Each question was rated on a 5-point Likert scale, with high total scores indicating better COVID-19-related crisis communication. The reliability of the tool showed a Cronbach’s α value of 0.788 in the present study. 

#### 2.3.4. COVID-19 Health Literacy 

COVID-19 health literacy was measured using the tool developed by Park et al. [28]. This tool consisted of 15 items on literacy regarding COVID-19 (i.e., active surveillance, self-isolation, secondary infection, route of COVID-19 virus infection, etc.). Content validity was verified by 12 infection-medical experts. As a result of the verification, the CVI of each question was 0.80 or higher, and the existing 15 questions were confirmed without modification or deletion. Each item was rated on a 4-point Likert scale, with higher total scores indicating higher COVID-19 health literacy. The reliability of the tool showed a Cronbach’s α value of 0.851 at the time of development [28] and a Cronbach’s α value of 0.917 in the present study.

#### 2.3.5. COVID-19 Infection Prevention Behaviors 

COVID-19 infection prevention behaviors were measured using a tool that was developed by Park et al. [28]. Content validity was verified by 12 infection-medical experts, and each item had a CVI of 0.80 or higher. This tool consisted of 13 items designed to assess the level of compliance for avoidance of private gatherings, using public transportation systems, and visiting COVID-19 outbreak areas and institutions; disinfection and ventilation; use of hand sanitizers; compliance with cough etiquette; and mask-wearing. Each item was rated on a 5-point Likert scale, with higher total scores indicating higher COVID-19 infection prevention behaviors. The reliability of the tool showed a Cronbach’s α value of 0.763 at the time of development and a Cronbach’s α value of 0.866 in the present study.

### 2.4. Data Collection

The data collection period was between 29 July and 4 August 2020. After preparing an online questionnaire, consisting of information about the study and the survey questions, a preliminary survey was conducted on 30 workers in the automobile manufacturing sector in Korea to check for readability of the questionnaire and difficulties understanding any of the items, after which, the main survey was conducted using an online survey. After receiving information about the objectives and methods of the study and the voluntary nature of participation in the study, those who understood the goal of the study and voluntarily consented to participate in the study were instructed to complete the informed consent form (ICF) online before completing the questionnaire. The participants who completed the survey received a small token of appreciation.

### 2.5. Ethical Considerations

The present study was conducted after the necessary approval was obtained from the Institutional Review Board (IRB) of Woosuk University (IRB no. WS-2020-10). For the ethical protection of the participants, all participants were informed about the objectives and methods of the study, voluntary nature of participation in the study, the right to withdraw at any time, and no disadvantage due to nonparticipation. Those who voluntarily signed an ICF to participate in the online questionnaire survey were included in the study. Personally identifiable information, such as name and telephone number, was not collected. The collected data were encrypted and stored on a password-protected personal computer.

### 2.6. Analysis Method

The data were analyzed using SPSS/WIN 25.0. Frequency and descriptive statistics analyses were performed to identify the general characteristics of the participants. Descriptive statistical analysis was performed to identify COVID-19 risk perception, crisis communication, health literacy, and infection prevention behaviors. To test whether there are significant differences in COVID-19 risk perception, crisis communication, health literacy, and infection prevention behaviors according to the general characteristics of the participants, independent sample t-test and one-way analysis of variance (ANOVA) were performed. Scheffé post-hoc test was performed on variables that showed a significant difference. Pearson’s correlation analysis was performed to identify the correlations among COVID-19 risk perception, crisis communication, health literacy, and infection prevention behaviors. Multiple regression analysis was performed to identify the influencing factors of COVID-19 infection prevention behaviors.

## 3. Results

### 3.1. Participant’s Characteristics

The general characteristics of the participants are shown in Table 1. There were 129 males (82.2%) and 28 females (17.8%), and there were 83 participants (52.9%) aged 20–39 years and 74 participants (47.1%) aged ≥ 40 years. With respect to marital status, 55 participants (35.0%) were single, and 102 participants (65.0%) were married. With respect to education level, 85 participants (54.1%) were college graduates, 61 participants (38.9%) were high school graduates, and 11 participants (7.0%) were graduate school graduates or higher. With respect to employment type, 132 participants (84.1%) were regular workers, and 25 participants (15.9%) were contract workers. Length of employment at current job appeared in the order of ≥10 years (n = 77, 49.0%), followed by <5 years (n = 51, 32.5%), and 5 to <10 years (n = 29, 18.5%). Subjective economic status appeared in the order of middle (n = 132, 84.1%), followed by lower (n = 17, 10.8%), and upper (n = 8, 5.1%). 

With respect to COVID-19 prevention education experience, 124 participants (79.0%) responded that they had such experience. For the source of COVID-19 information, the participants were allowed to choose more than one response, and the responses included company (n = 86, 54.8%), KDCA or health center (n = 58, 36.9%), and mass media (n = 115, 73.2%). With respect to subjective health status, 142 participants (90.4%) responded that they were healthy, and 15 participants (9.6%) responded that they were unhealthy. There was one participant (0.6%) who was confirmed to have been infected with COVID-19, while one participant (0.6%) had been placed under self-isolation. One participant (0.6%) had a cohabiting family member with confirmed COVID-19, while 41 participants (26.1%) had cohabiting family members with chronic diseases, such as hypertension, diabetes, and cancer.

### 3.2. COVID-19 Risk Perception, Crisis Communication, Health Literacy, and Infection Prevention Behaviors

The results regarding COVID-19 risk perception, crisis communication, health literacy, and infection prevention behaviors of the participants are shown in Table 2. The mean scores for COVID-19 risk perception was 3.43 ± 0.66 points (range: 1–5 points); COVID-19 crisis communication was 3.87 ± 0.64 (range: 1–5 points); COVID-19 health literacy was 3.31 ± 0.40 points (range: 1–4 points); and COVID-19 infection prevention behaviors was 4.17 ± 0.49 points (range: 1–5 points). 

With respect to sub-items of COVID-19 infection prevention behaviors, the mean scores were high for “avoidance of COVID-19 outbreak institution” (4.42 ± 0.73 points) and “mask wearing” (4.36 ± 0.62 points), whereas the mean scores were low for “disinfection of areas frequently touched by hand” (3.73 ± 0.83 points) and “ventilation” (3.82 ± 0.86 points) (Table 3).

### 3.3. Differences in COVID-19 Risk Perception, Crisis Communication, Health Literacy, and Infection Prevention Behaviors According to the General Characteristics

Differences in COVID-19 risk perception, crisis communication, health literacy, and infection prevention behaviors according to the general characteristics of the participants are shown in Table 4. 

There were significant differences in COVID-19 risk perception according to cohabiting with a family member with a chronic disease (t = 2.17, *p* = 0.03), with higher COVID-19 risk perception among those with a cohabiting family member with a chronic disease than those without. 

There were significant differences in COVID-19 crisis communication according to age (F = 0.82, *p* = 0.044), education level (F = 8.25, *p* < 0.001), subjective economic status (F = 5.30, *p* = 0.006), COVID-19 prevention education (t = 3.79, *p* < 0.001), information acquisition through the company (t = 2.96, *p* = 0.004), and subjective health status (t = 2.88, *p* = 0.005). Participants aged 20–39 years had education levels reading graduate school graduates or higher and had a subjective economic status of upper. They also had higher levels of crisis communication than their counterparts. Moreover, those who received COVID-19 prevention education, did not acquire COVID-19 information through their company, and perceived themselves to be healthy, had higher levels of crisis communication. 

There were significant differences in COVID-19 health literacy according to sex (t = −3.30, *p* < 0.001), education level (F = 11.22, *p* < 0.001), subjective economic status (F = 6.26, *p* = 0.002), and COVID-19 prevention education (t = 2.18, *p* = 0.031). Participants who were females, had education levels reading graduate school graduates or higher, had a subjective economic status of upper, and had higher levels of health literacy than their counterparts. Moreover, those who received COVID-19 prevention education and did not acquire COVID-19 information through their company had higher levels of health literacy.

There were significant differences in COVID-19 infection prevention behaviors according to the length of employment at current job (F = 0.30, *p* = 0.045), COVID-19 prevention education (t = −1.17, *p* = 0.025), and information acquisition through the KDCA/health center (t = 2.45, *p* = 0.015). Participants with an employment period at current job of 5 to <10 years, received COVID-19 prevention education, acquired COVID-19 information through the KDCA/health center and had higher levels of infection prevention behaviors than their counterparts.

### 3.4. Correlations among COVID-19 Risk Perception, Crisis Communication, Health Literacy, and Infection Prevention Behaviors

The correlations among COVID-19 risk perception, crisis communication, health literacy, and infection prevention behaviors are shown in Table 5. COVID-19 risk perception showed significantly positive correlations with crisis communication (r = 0.163, *p* = 0.04) and infection prevention behaviors (r = 0.213, *p* = 0.007). COVID-19 crisis communication showed a significantly positive correlation with COVID-19 health literacy (r = 0.529, *p* < 0.001). COVID-19 infection prevention behaviors showed significantly positive correlations with risk perception (r = 0.213, *p* = 0.007), crisis communication (r = 0.374, *p* < 0.001), and health literacy (r = 0.423, *p* < 0.001).

### 3.5. Influencing Factors of COVID-19 Infection Prevention Behaviors

The results from the analysis of the influencing factors of COVID-19 infection prevention behaviors of workers are shown in Table 6. To check whether the basic assumptions of regression analysis were satisfied before the analysis, as a result of checking the P-P diagram and the scatterplot, the normality of the residuals was satisfied as it was close to a 45° straight line, and the residuals were all evenly distributed around zero, thus, satisfying the linearity and the assumption of equal variance of the model. As a result of examining multicollinearity, the tolerance value of the variables was 0.30~0.52, which was more than 0.1, and the value of the variance inflation factor was 1.70~2.08, which did not exceed 10, so it was a problem of multicollinearity between independent variables. The VIF value is 1 point, much smaller than 10, so there is no multicollinearity problem.

General characteristics that showed significant differences in infection prevention behaviors (length of employment at current job and source of information) and the independent variables of the study (COVID-19 risk perception, crisis communication, and health literacy) were inputted. 

The regression model was found to be significant (F = 12.5, *p* < 0.001), while employment period at current job, source of information, COVID-19 risk perception, crisis communication, and health literacy were also found to be significant. Among the demographic variables, employment period at current job of 5~10 years (β = 0.19, *p* = 0.01) showed higher levels of infection prevention behaviors than that of <5 years. The level of infection prevention behaviors was also significantly higher when COVID-19 prevention education was experienced (β = 0.20, *p* = 0.004).

Moreover, the level of infection prevention behaviors was also significantly higher when COVID-19-related information was acquired through the KDCA/health center (β = 0.17, *p* = 0.01). Higher COVID-19 risk perception (β = 0.22, *p* = 0.002), crisis communication (β = 0.21, *p* = 0.013), and health literacy (β = 0.31, *p* < 0.001) were associated with significantly higher levels of infection prevention behaviors.

## 4. Discussion

Workers who work face-to-face at a time when infectious diseases, such as COVID-19 are prevalent may be vulnerable to infection [29]. Therefore, the present study identified the levels of risk perception, crisis communication, health literacy, and infection prevention behaviors of workers who are not given access to flexible working conditions in the automobile manufacturing sector, in addition to the influencing factors of infection prevention behaviors, with the objective of providing basic data for developing infectious disease prevention programs for workers in the manufacturing sector.

The findings of this study showed that the mean score for COVID-19 infection prevention behaviors was 4.17 points, and the influencing factors of infection prevention behaviors were identified to be COVID-19 health literacy, risk perception, length of employment at current job, crisis communication, and source of information in order.

In the present study, the mean score for COVID-19 infection prevention behaviors of workers in the automobile manufacturing sector was 4.17 out of 5 points. This was a relatively higher score than the median score of 13.5 points (range: 0–19) reported by a study on adults in Mexico City [30] using a different scale. Among the sub-items of infection prevention behaviors, “avoidance of COVID-19 outbreak institution,” “compliance with cough etiquette,” and “mask wearing” showed high scores, whereas “disinfection of surrounding environment” and “ventilation” showed low scores. Such findings share a similar context as a study that reported healthcare workers who treated face-to-face treatments practiced the preventive behaviors of not visiting COVID-19 outbreak areas and avoiding contact with other people the most [31]. During the early stages of COVID-19, when the study was conducted, COVID-19 vaccines and therapeutics were being developed, while the WHO was emphasizing nonpharmaceutical interventions, such as hand washing, mask-wearing, and social distancing [4]. Therefore, it is believed that such results could be attributed to why business owners are focusing on environmental disinfection and ventilation to prevent the worst-case scenario of a business shut-down due to a mass infection outbreak. Workers in the manufacturing sector personally practice preventive behaviors as best they can within the possible scope of their job characteristics, as the environment of manufacturing requires face-to-face engagement on site, which makes social distancing difficult. Moreover, KDCA implemented mandatory mask-wearing, which promotes the concept that “mask wearing is the easiest and most reliable vaccine.” Consequently, compliance with mask-wearing was high. Currently, KDCA has been recommending vaccine booster shots and urging continued practice of preventive behaviors in response to the prolongation of COVID-19 and the emergence of new variants, and as a result, workers are experiencing a gradual increase in fatigue [32]. Nonetheless, personal infection prevention behaviors, such as mask-wearing, are one of the disease control measures with the lowest social cost [33]. Therefore, it is necessary to identify items that are undervalued in infection prevention behavior, check the cause, and educate them to convey correct knowledge and importance so that infection prevention behavior can be habituated.

In the present study, COVID-19 health literacy was the biggest influencing factor of infection prevention behaviors, where higher health literacy was correlated with a higher level of practicing infection prevention behaviors. A study conducted in Sydney on adults reported that people with low health literacy had a higher risk of infection and mortality because they were less capable of understanding the symptoms of COVID-19 and identifying infection prevention behaviors [34]. Moreover, another study on Japanese adults also reported that health literacy influenced COVID-19 infection prevention behaviors [35]. Consequently, differences in health literacy between individuals can lead to the transmission of COVID-19 to others, and thus, enhancing health literacy in individuals for the prevention of COVID-19 is an important part of healthcare for the worker groups. Furthermore, health literacy must be based on skills that assess a broad range of information and allow making appropriate decisions [35], and thus, reliable information that can be easily understood must be provided to workers, such as contents that give access to information assessment and decision-making for enhancing health literacy when conducting safety and conducting health education mandated by the Industrial Safety and Health Act.

The findings of the study also showed that COVID-19 risk perception is a major influencing factor in infection prevention behaviors. Such findings supported the results from previous studies indicating that perceived risk was a key factor in inducing behavioral change [7] and that higher risk perception was associated with practicing infection prevention behaviors against H1N1 influenza [16] and COVID-19 [17,18]. Moreover, COVID-19 risk perception was found to have an influence on preventive behavior intentions by mediating attitudes, subjective norms, and perceived behavioral control [17]. People who have a high perception of the seriousness of COVID-19 and vulnerability to infection tend to maintain vigilance and take aggressive actions [18], and thus, the health managers at businesses should consider this aspect to keep workers safe during the COVID-19 pandemic.

In the present study, more active crisis communication was correlated with higher levels of practicing infection prevention behaviors, which was consistent with previous studies reporting that sufficient information exchange or acquisition is associated with practicing healthy behaviors [8,19,20]. Moreover, active crisis communication can also help determine the attitudes, subjective norms, and risk perceptions regarding preventive behaviors [17]. Effective information exchange and communication about infectious diseases during a crisis can increase the understanding of the health risk of that infectious disease and help with decision-making for mitigating the risk of promoting changes in human behavior and can, thus, be a key strategy for achieving public health goals [36]. Furthermore, a previous study reported that providing information through advertising communication during COVID-19 quarantine increases psychological resilience and helps relieve stress [37]. Based on this, if the state provides sufficient communication, including information on the importance of infection prevention actions, we expect that it will be possible to not only alleviate the stress of the subjects due to the infectious disease situation, but also promote preventive actions.

The employment period at a current job was also identified as an influencing factor of infection prevention behaviors. Workers with 5~10 years of experience at their current job had significantly higher levels of infection prevention practices than those with <5 years of experience at their current job. In a study on the infection prevention behaviors of workers in tuberculosis specialty hospitals [38], workers with ≥5 years of tuberculosis hospital experience showed a higher level of practicing tuberculosis preventive behaviors than those with <5 years of experience, and the same result was found in a study on health care providers in Yemen [39]. These results are thought to be the effects obtained through experience of responding to infectious diseases, such as H1N1 influenza and MERS, and education on prevention of infectious diseases while working. Therefore, in health and safety education, education that promotes appropriate infection prevention behavior suitable for job characteristics should be strengthened in consideration of work experience.

The workers with COVID-19 prevention education had significantly higher levels of infection prevention practices. Workers who have received COVID-19 infection prevention education will have a higher level of knowledge about COVID-19. It is consistent with the results of previous studies [35,38,39], which state that the higher the level of knowledge, the higher the performance of infection prevention actions. Therefore, it is necessary to provide consistent health information education in order to increase access to health information at work.

Lastly, those who acquired COVID-19-related information through the KDCA/health center showed significantly higher levels of infection prevention practices. A study conducted in China reported that individuals who had a high interest in COVID-19 information through the official state media were 4.16 times more likely to practice infection prevention behaviors than those with a low interest in such information. This suggests that maintaining public trust with respect to information announced by government agencies is also an important factor [18]. Currently, there is a lot of health-related information circulating through various media outlets and internet-based media in Korea, resulting from advanced internet technology and a heightened interest in health. However, during a pandemic situation, such as what people are facing now, there is the concern of spreading the infection through improper behaviors stemming from the infodemic [40]. In a study on Portuguese university students, the results showed that individuals who used websites of public agencies to seek information had higher COVID-19-related digital health literacy than those using social media for such information [41]. Therefore, managers in charge of workplace safety and health should provide credible and up-to-date data, such as response guidelines from KDCA, health centers, and the Ministry of Employment and Labor to the workers in a timely manner and also prepare platforms in the workplace to increase the participation and satisfaction of workers, so that fear of COVID-19 due to false information can be reduced and infection prevention behaviors can be enhanced. Additionally, to ensure the effectiveness of warnings, messages issued by managers in charge of workplace safety and health should be comprehensible, concise, and convincing [42].

Adherence to these guidelines can be affected by many factors, including how these messages are communicated and the attitudes of the individuals [43]. In this context, it is fundamental that managers in charge of workplace safety and health convey the right messages, in the right way, to their workers.

This study is a cross-sectional study, and there is a limit on generalizing the results of the study because the evidence for causality is weak and also because data was conveniently extracted from workers in partial areas of the automobile manufacturing industry. Additionally, in the case of the automobile manufacturing industry, there is a large difference in distribution between men and women due to the nature of the work, and job characteristics include harmful factors in the working environment. Therefore, a follow-up study considering gender distribution and harmful factors in manufacturing is needed in future studies on manufacturing workers. Finally, this study used an instrument that tested only content validity and reliability for rapid research in public health emergency situations. Therefore, further studies on the development of a valid and reliable instrument to measure variables related to the prevention behavior of new infectious diseases are necessary for the consideration of the target characteristics.

## 5. Conclusions and Recommendations

The present study was a descriptive survey study for identifying the influencing factors of COVID-19 preventive behaviors of workers in the manufacturing sector. The findings showed that the mean score for COVID-19 preventive behaviors was 4.17 points, and the influencing factors identified were: employment period at current job, COVID-19 prevention education, source of information, risk perception, crisis communication, and health literacy.

With the continuation of the COVID-19 pandemic, experts are warning about the risk of new infectious diseases that may emerge in the future. Due to the nature of their work, workers in the manufacturing sector face the risk of infection due to difficulties in maintaining social distancing, and if infected, they also have a high risk of spreading the infection to family members and the community at large. Moreover, a mass infection outbreak at a workplace could result in the suspension or closure of businesses, which could have a very significant ripple effect on the national economy. Therefore, the following practical recommendations are made based on the findings of the present study.

Although the employment rate in the service industry has plummeted due to COVID-19, the demand for workers in the manufacturing sector has increased as production increases in the automobile, machinery, and electronics industries. Even if an emerging infectious disease spreads, in order to maintain and grow the economy, individual efforts to prevent infection are required in addition to the efforts of the government and business owners. Therefore, based on the results, health managers need to develop programs and educate people so as to improve information comprehension and crisis communication in order to promote workers’ infection prevention behavior of emerging infectious diseases in an era of global change.

## Figures and Tables

**Table 1:** General characteristics of the participants’ variables (N = 157).

Variable		n	%	M ± SD
Sex	Male	129	82.2	
	Female	28	17.8	
Age (years)	20–39	83	52.9	39.29 ± 11.27
	≥40	74	47.1	
Marital status	Single	55	35.0	
	Married	102	65.0	
Education level	High school	61	38.9	
	College	85	54.1	
	Graduate school	11	7.0	
Employment type	Regular	132	84.1	
	Contract	25	15.9	
Period of employment at current job (years)	<5	51	32.5	12.45 ± 10.9
	5–10	29	18.5	
	≥10	77	49.0	
Subjective economic status	Upper	8	5.1	
	Middle	132	84.1	
	Lower	17	10.8	
COVID-19 infection prevention education	Yes	124	79.0	
	No	33	21.0	
Source of COVID-19 information (more than one response possible)	Company	86	54.8	
	KDCA/health center	58	36.9	
	Mass media	115	73.2	
Subjective health status	Healthy	142	90.4	
	Unhealthy	15	9.6	
Cohabiting with a family member with a chronic disease	Yes	41	26.1	
	No	116	73.9	

**Table 2:** Descriptive statistics of COVID-19-releated variables (N = 157).

Variable	Possible Range	M ± SD	Skewness	Kurtosis
Risk perception	1–5	3.43 ± 0.66	−0.32	1.17
Crisis communication	1–5	3.87 ± 0.64	0.04	−0.10
Health literacy	1–4	3.31 ± 0.40	0.05	−1.02
Infection prevention behaviors	1–5	4.17 ± 0.49	−0.52	1.41

**Table 3:** Descriptive statistics of infection prevention behavior about COVID-19 (N = 157).

Items	M ± SD (Range 1~5)
1. Avoidance of private gathers	4.13 ± 0.67
2. Avoidance of public transportation system	4.28 ± 0.68
3. Avoidance of visiting crowded places	4.08 ± 0.71
4. Avoidance of visiting COVID-19 outbreak area	4.36 ± 0.79
5. Avoidance of visiting COVID-19 outbreak institution	4.42 ± 0.73
6. Social distancing in daily life	4.11 ± 0.66
7. Disinfection of areas frequently touched by hand	3.3.73 ± 0.83
8. Ventilation	3.3.82 ± 0.86
9. Use of hand sanitizer	4.4.34 ± 0.66
10. Compliance with cough etiquette	4.4.36 ± 0.64
11. Mask-wearing	4.4.36 ± 0.62
12. Refrain from going outside if fever or respiratory symptoms are present	4.4.30 ± 0.65
13. Not touching eyes or nose with unwashed hands	3.3.97 ± 0.79
Total	4.17 ± 0.49

**Table 4:** COVID-19 risk perception, crisis communication, COVID-19 health literacy, and infection prevention behaviors according to the general characteristics (N = 157).

Variable		n	Risk Perception		Crisis Communication		Health Literacy		Infection Prevention Behaviors	
			M ± SD	t/F (*p*)	M ± SD	t/F (*p*)	M ± SD	t/F (*p*)	M ± SD	t/F (*p*)
Sex	Male	129	3.44 ± 0.68	0.68 (0.50)	3.84 ± 0.63	0.66 (0.183)	3.27 ± 0.39	−3.30 (<0.001) **	4.16 ± 0.50	−0.70 (0.487)
	Female	28	3.36 ± 0.52		4.02 ± 0.65		3.54 ± 0.38		4.23 ± 0.48	
Age (years)	20–39 ^a^	83	3.42 ± 0.61	0.57 (0.57)	3.93 ± 0.67	0.82 (0.044) * b, a < c	3.34 ± 0.42	1.61 (0.204) a, b < c	4.14 ± 0.48	1.87 (0.157)
	40–59 ^b^	71	3.43 ± 0.70		3.89 ± 0.58		3.28 ± 0.38		4.20 ± 0.50	
	≥60 ^c^	3	3.83 ± 0.76		3.83 ± 1.04		3.67 ± 0.58		4.67 ± 0.58	
Marital status	Single	55	3.48 ± 0.57	0.68 (0.50)	3.95 ± 0.65	1.19 (0.237)	3.32 ± 0.42	0.11 (0.913)	4.14 ± 0.50	−0.66 (0.511)
	Married	102	3.41 ± 0.70		3.83 ± 0.62		3.31 ± 0.39		4.19 ± 0.49	
Education level	High school ^a^	61	3.51 ± 0.69	1.02 (0.36) c < a	3.67 ± 0.58	8.25 (<0.001) *** a < c	3.19 ± 0.36	11.22 (<0.001) ** a < c	4.11 ± 0.44	2.64 (0.075)
	College ^b^	85	3.41 ± 0.64		3.94 ± 0.64		3.36 ± 0.39		4.18 ± 0.52	
	Graduate school	11	3.22 ± 0.66		4.41 ± 0.54		3.74 ± 0.33		4.48 ± 0.51	
Employment type	Regular	132	3.46 ± 0.67	1.11 (0.27)	3.85 ± 0.63	−0.92 (0.358)	3.30 ± 0.40	−1.56 (0.121)	4.17 ± 0.50	−0.55 (0.582)
	Contract	25	3.30 ± 0.52		3.98 ± 0.67		3.43 ± 0.41		4.22 ± 0.47	
Period of employment at current job (years)	<5 ^a^	51	3.38 ± 0.46	0.26 (0.77) a < b, c	3.97 ± 0.63	2.45 (0.090)	3.35 ± 0.39	0.301 (0.741)	4.07 ± 0.45	0.30 (0.045) * a < b
	5~10 ^b^	29	3.43 ± 0.87		4.00 ± 0.69		3.31 ± 0.48		4.36 ± 0.47	
	≥10 ^c^	77	3.47 ± 0.68		3.76 ± 0.61		3.30 ± 0.38		4.18 ± 0.51	
Subjective economic status	Upper ^a^	8	3.31 ± 0.46	0.32 (0.73)	4.57 ± 0.56	5.30 (0.006) * b, c < a	3.79 ± 0.29	6.26 (0.002) ** c < b < a	4.46 ± 0.52	1.45 (0.239) b, c < a
	Middle ^b^	132	3.42 ± 0.67		3.83 ± 0.61		3.29 ± 0.39		4.16 ± 0.49	
	Lower ^c^	17	3.53 ± 0.62		3.88 ± 0.70		3.29 ± 0.39		4.15 ± 0.44	
COVID-19 infection prevention education	Yes	124	3.41 ± 0.65	−0.81 (0.14)	3.97 ± 0.61	3.79 (<0.001) ***	3.35 ± 0.40	2.18 (0.031) *	4.15 ± 0.49	−1.17 (0.025) *
	No	33	3.52 ± 0.69		3.52 ± 0.61		3.18 ± 0.40		4.26 ± 0.49	
Source of COVID-19 information (more than one response possible)	Company	86	3.36 ± 0.63	−1.54 (0.13)	4.00 ± 0.66	2.96 (0.004) **	3.37 ± 0.42	1.76 (0.078)	4.22 ± 0.47	1.29 (0.198)
	KDCA/health center	58	3.30 ± 0.71	−1.94 (0.05)	3.98 ± 0.64	1.67 (0.096)	3.35 ± 0.42	0.895 (0.372)	4.30 ± 0.42	2.45 (0.015) *
	Mass media	115	3.46 ± 0.64	0.88 (0.38)	3.89 ± 0.63	0.47 (0.641)	3.34 ± 0.38	1.26 (0.209)	4.19 ± 0.50	0.61 (0.543)
Subjective health status	Healthy	142	3.41 ± 0.67	−1.25 (0.22)	3.92 ± 0.63	2.88 (0.005) **	3.33 ± 0.41	0.88 (0.382)	4.18 ± 0.50	0.26 (0.797)
	Unhealthy	15	3.60 ± 0.52		3.43 ± 0.53		3.23 ± 0.31		4.14 ± 0.41	
Cohabiting with a family member with a chronic disease	Yes	41	3.52 ± 0.58	2.17 (0.03) *	3.91 ± 0.66	0.492 (0.624)	3.31 ± 0.42	−0.10 (0.92)	4.24 ± 0.43	0.99 (0.323)
	No	116	3.36 ± 0.67		3.86 ± 0.63		3.32 ± 0.40		4.15 ± 0.51	

* *p* < 0.05, ** *p* < 0.01, *** *p* < 0.001.

**Table 5:** Correlations among COVID-19-related variables (N = 157).

Variable	1	2	3	4
	r (*p*)			
1. Risk perception	1			
2. Crisis communication	0.163 (0.04) *	1		
3. Health literacy	0.070 (0.41)	0.529 (<0.001) ***	1	
4. Infection prevention behaviors	0.213 (0.007) ***	0.374 (<0.001) ***	0.423 (<0.001) ***	1

* *p* < 0.05, *** *p* < 0.001.

**Table 6:** Influencing factors of COVID-19 infection prevention behaviors (N = 157).

Variable	B	t	SE	β	*p*
(Constant)	1.33	4.01	0.33		<0.001
Period of employment at current job [1] (years)					
5–10	0.24	2.52	0.09	0.19 *	0.01
≥10	0.08	1.11	0.08	0.09	0.27
COVID-19 prevention education [2]					
Yes	0.24	2.90	0.08	0.20 **	0.004
Source of information [3]					
KDCA/health center	0.18	2.60	0.07	0.17 *	0.01
Risk perception	0.07	3.20	0.02	0.22 **	0.002
Crisis communication	0.16	2.52	0.06	0.21 *	0.013
Health literacy	0.38	4.07	0.09	0.31 **	<0.001

F = 12.5 (*p* < 0.001), R^2^ = 0.370, adj R^2^ = 0.340. * *p* < 0.05, ** *p* < 0.01, Ref: [1] Period of employment at current job (years) < 5; [2] COVID-19 prevention education no.; [3] KDCA/health center use no.

## Data Availability

Not applicable.
